# Tephra fallout hazard assessment for a hydrovolcanic eruptive scenario in Mayotte

**DOI:** 10.1038/s41598-024-83266-x

**Published:** 2024-12-30

**Authors:** Audrey Michaud-Dubuy, Jean-Christophe Komorowski, Tristan Lacombe, Lucia Gurioli

**Affiliations:** 1 Clermont Auvergne University, CNRS, IRD, OPGC, Magmas and Volcanoes Laboratory, 63000 Clermont-Ferrand, France; 2 Institute of Earth Physics of Paris (IPGP), Paris Cité University, CNRS, Paris, France

**Keywords:** Geology, Geophysics, Volcanology, Natural hazards

## Abstract

The new submarine volcano Fani Maoré offshore Mayotte (Comoros archipelago) discovered in 2019 has raised the awareness of a possible future eruption in Petite-Terre island, located on the same 60 km-long volcanic chain. In this context of a renewal of the volcanic activity, we present here the first volcanic hazard assessment in Mayotte, focusing on the potential reactivation of the Petite-Terre eruptive centers. Using the 2-D tephra dispersal model HAZMAP and the 1979 $-$ 2021 meteorological ERA-5 database, we first identify single eruptive scenarios of various impacts for the population of Mayotte. Even when considering the least impacting scenario, we show that ~ 30,000 people could be threatened by a future explosive eruption in the highly densely populated island of Petite-Terre. We then use a Monte Carlo approach to sample a series of eruptive scenarios and produce a probabilistic map allowing a long-term vision of the tephra fallout hazard in Mayotte. Finally, we discuss the probability of the different eruptive scenarios based on new field data and show that both Mamoudzou (Grande-Terre) and Petite-Terre could be impacted by at least 5 to 40 cm of tephra. These crucial results will be included in Mayotte’s first volcano emergency plan.

## Introduction

**Fig. 1:**
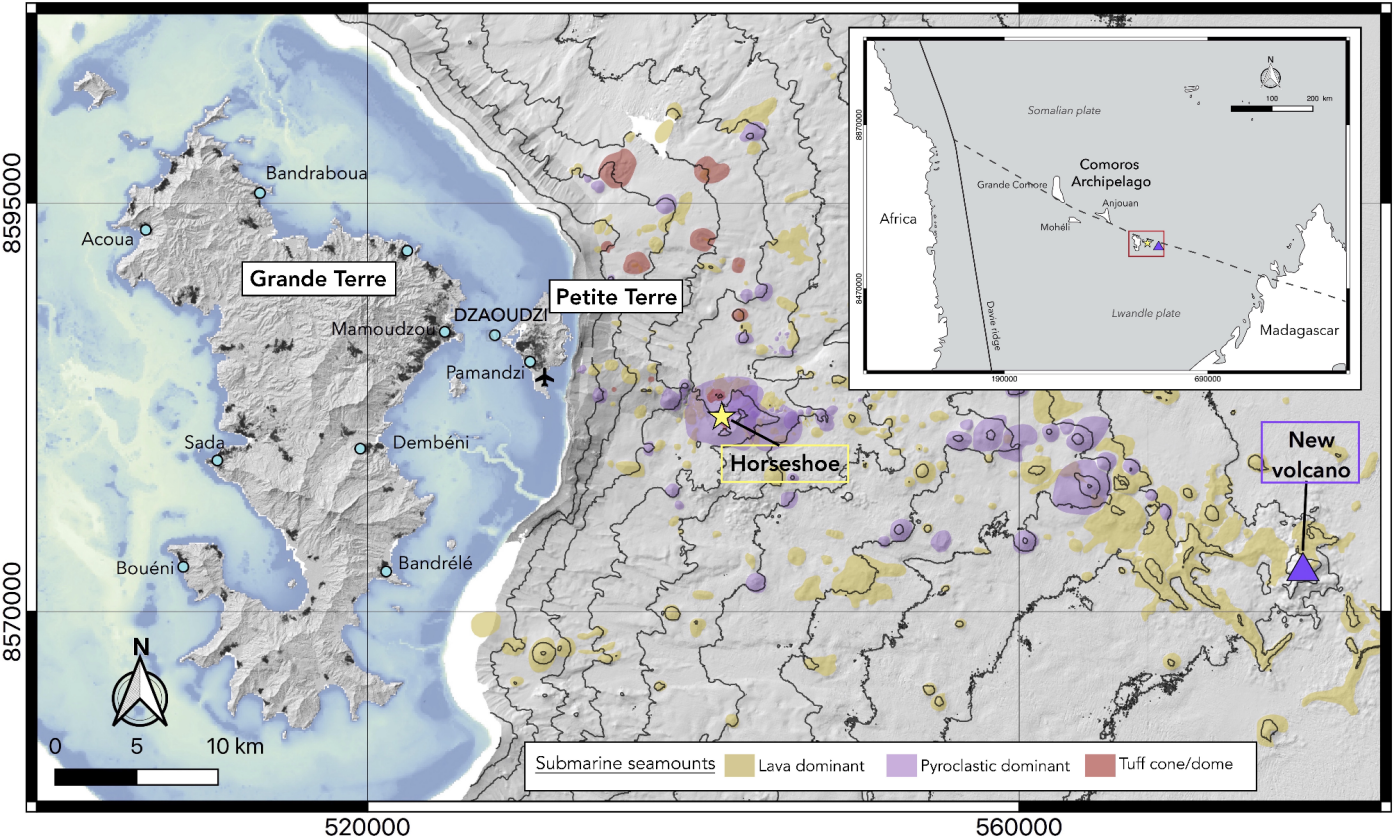
Geological context of the study with the location of Mayotte in the Mozambique channel (red rectangle in inset). The main map shows the location of the new volcano Fani Maoré, about 50 km away from Mayotte (purple triangle), the “horseshoe” area characterized by high seismic activity and fluid emissions (yellow star), older volcanic cones (modified from^16^) in yellow (lava dominant), purple (pyroclastic dominant) and red (tuff cone or dome), the main cities and villages of the island (blue circles), and the Pamandzi international airport on Petite-Terre. All maps in Figures 1 to 6 were generated by the authors with the open-source software QGIS (version 3.10.10, https://www.qgis.org/), the coordinates are in WGS 84 – UTM Zone 38S.

In May 2019, the first of a series of oceanographic cruises organized by the French scientific community (MAYOBS1^1^^[,2^) discovered a new submarine volcano, named Fani Maoré, 50 km off the eastern coast of Mayotte (purple triangle in Fig. 1^3,4^). The birth of this 820 m-high cone located at a depth of 3300 m^4–6^has been at the origin of the seismic crisis recorded in Mayotte Island (Comoros archipelago) in May 2018^7,8^. Since September 2018, two seismic clusters located between 5 and 15 km east of Petite-Terre (at mantle depths, > 30 km) and at 25 km east of Petite-Terre (25–50 km deep) have been active. They are thought to be associated with the deformation of two magma reservoirs and to dike propagation, respectively^4,9–11^, which is consistent with petrological and geobarometry data on eruptive products^5^. Several events were felt by the population in Mayotte (e.g., the M_w_ 5.9 earthquake on May 15, 2018, or the last felt ML 4.9 event on August 27, 2024^4^^[,12^). Since December 4, 2020^13^, seismic activity and fluid emissions are still recorded in the horseshoe area (^12^, Fig. 1). The latter includes acoustic plumes of hydrothermal or magmatic nature in the water column above active lava flows^14^, plumes of liquid CO_2_with a magmatic mantle isotopic signature^15^ within and near the horseshoe-shaped collapse structure located in an area of numerous overlapping pyroclastic cones, domes and lava flows atop the main seismic cluster (yellow star in Fig. 1), as well as CO_2_ and CH_4_ gaseous emissions on Petite-Terre^12^.

The Fani Maoré eruption demonstrates that the Comoros archipelago (composed of four EW aligned islands and located north of the Mozambique Channel in the Indian ocean, see inset in Fig. 1) is still volcanically active. The cause of the volcanic activity in this region is a long-lasting debate, but latest findings suggest that the lithospheric deformation related to East African Rift dynamics (rather than the interaction of a mantle plume with the oceanic lithosphere) is most likely the source of the volcanism^5,8,17–20^. Mayotte, the easternmost and oldest island of the archipelago, is composed of two main volcanic islands: Grande-Terre and Petite-Terre. The volcanic activity in Mayotte dates mainly from the Mio-Pliocene, but recent Holocene eruptive centers were dated east of Grande-Terre and built Petite-Terre^17,18,21–25^. This volcanism takes place along a WNW-ESE trending mainly submarine volcanic chain and stretching from the Mamoudzou area on Grande-Terre to 60 km offshore Mayotte to the East (i.e., towards Fani Maoré, see Fig. 1^4,6,20^;). Petite-Terre stands on the reef barrier and is composed of the most recent volcanic products of Mayotte^24^: scoria cones resulting from eruptions of basanitic and tephritic lavas, and four main phonolite explosive edifices originally dated at 6 $-$7 ka^26^, but probably occurred earlier during the last glacial age before 25 ka BP^27^. The emitted magmas along the chain are indeed characterized by a bimodal chemical distribution of basanite and phonolite with rare intermediate compositions^16^, which formed lava flows, domes, scoria, pumice and tuff cones, maars, and eruptive fissures (Fig. 1). Basanitic magmas are first stored and evolve in deep magma reservoirs located between 35 and 60 km depth^16^. They can then either rise directly to the surface (e.g., the 2018–2020 eruption), pause in the shallower reservoir (an extensive, long-lasting crustal mush magmatic system highlighted by clinopyroxene barometry) before resuming its ascent, or stop and evolve to phonolite in the shallower reservoir^16^. This magmatic system most probably connects to the surface in the horseshoe area where bathymetry studies revealed more than 300 submarine cones and vents, but also branches out to the west, towards Petite-Terre recent volcanic centers^6,16,27^. Low-temperature, CO_2_ and CH_4_ rich fluid emissions, as well as several areas of passive degassing of CO_2_, attest to a hydrothermal activity on Petite-Terre^4^.

In this context of reactivation of the whole magmatic system, the scenario of a potential future subaerial non-magmatic to magmatic eruption on Petite-Terre cannot be ruled out^6,27^. Such a scenario, in the highly densely populated area of Dzaoudzi-Mamoudzou (1600–2600 inhabitants/km^2^^28^,) could be catastrophic, and it is therefore crucial to investigate its impacts on Petite-Terre and Grande-Terre. Recent field-based studies on Petite-Terre allowed to better picture the formation of the four phonolitic explosive edifices of La Vigie, Moya, Central Crater, and Dziani^23,27^. The eruptive products display signs of a primary purely magmatic fragmentation (about 1 km deep), and then of a wet fragmentation, thus demonstrating a late interaction between magma and water close to the surface^27^. In this study, we thus considered an explosive eruption as the most likely scenario in case of a future eruption in Petite-Terre, with a possible late interaction with surface water (sea water or water contained in a crater lake in this case). This phenomenon usually results in a series of hazards similar to those of purely magmatic eruptions, i.e., tephra fall resulting from the formation of a volcanic column rising into the atmosphere, pyroclastic density currents (PDC), gas plumes, and/or lahars, but affecting a limited area surrounding the explosive vent(s). These “hydrovolcanic” eruptions have been much less studied than other eruption types. They are also rarely included in hazard assessment studies, even if they can be deadly and/or powerful (e.g., the 2014 Mt. Ontake and the 2019 Whakaari/ White Island eruptions^29,30^).

To characterize the tephra fallout hazard for an explosive hydrovolcanic scenario on Petite-Terre, we use the 2-D tephra dispersal model HAZMAP^31^whose input parameters consist of precise eruptive scenarios and atmospheric constraints (i.e., wind fields). Such models are indeed commonly used in the literature to produce volcanic hazard maps^32,33^. As this is the first hazard assessment for tephra fallout in this region, we follow the two most-used methodologies in the literature^32^: (1) the use of a single scenario based on a geological or conceptual case, and (2) a probabilistic approach with a quantitative estimate of the probabilities. We first identify several eruptive scenarios of various impacts on the population based on an analysis of the regional winds (using the European Centre for Medium-Range Weather Forecasts ERA5 reanalysis^34^). We then use a Monte Carlo approach to produce a probabilistic map allowing a broader vision of the tephra fallout hazard in Mayotte. Finally, we discuss the different eruptive scenarios considered in this study in light of new field-based data from^27^, and the potential impact of these scenarios on both population and critical infrastructures.

### Previous fieldwork in Petite-Terre

**Fig. 2:**
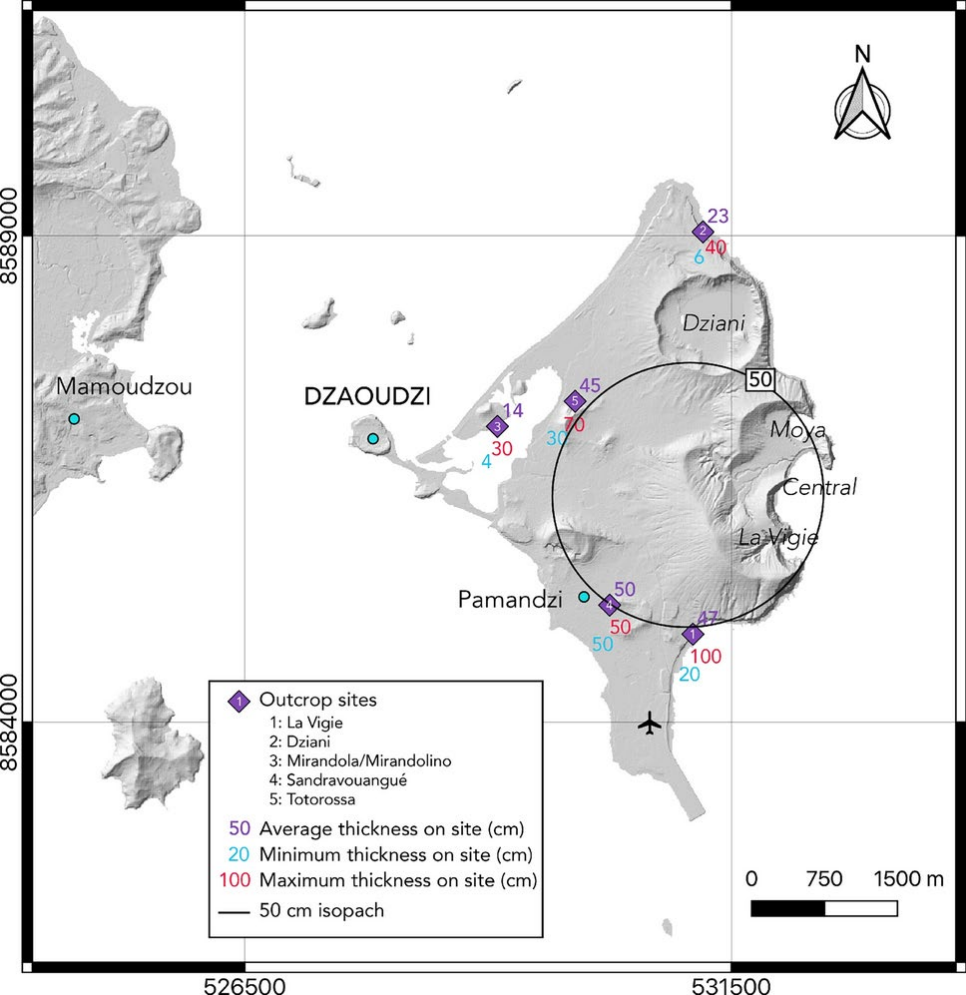
Deposit thickness measurements on Petite-Terre from^27^. From Table S1, we calculated for each outcrop sites (purple diamonds), the average, minimum and maximum fallout deposit thicknesses in centimeters (in purple, blue and red, respectively). The black circle represents the 50 cm isopach drawn from these measurements.

A new extensive field study of the four tuff rings and tuff cones of La Vigie, Moya, Central Crater, and Dziani in Petite-Terre was recently performed by^27^. Their study showed that they resulted from powerful explosive eruptions generating ballistic and tephra fallout together with fine ash pyroclastic density currents, for an approximate total volume of 0.24 km^3^DRE^27^. Five main sites were investigated by the authors, each site comprising several outcrops (purple diamonds in Fig. 2). At each outcrop, several deposits originating from different explosions/eruptive vents were visible (Figure S1). These deposits are usually well preserved and present alternations between fallout (Fa and Fb facies in Figure S1a, b and c), pyroclastic density currents (Pa, Pb, Ba, Bb facies in Figure S1c), and reworked (Rwk facies in Figure S1c) deposits. Because of the small area in which the fieldwork could have been performed (~ 12 km^2^), and the complex sedimentological features shown in Figure S1c and described in^27^, the authors will need further investigation before being able to reconstruct a precise eruptive chronology of the four events that built the phonolitic pyroclastic cones of Petite-Terre. We focus here on the fallout deposits Fa (Figure S1a, b and c). The Fa facies is usually massive with thicknesses ranging from 4 to 100 cm. The deposits are composed of well-sorted, clast-supported angular, lapilli-sized pumice and non-juvenile fragments, with neither grading nor lamination, which are typical features of air-fall deposits^27,35^. Their relative thinness together with the absence of grading suggest that these deposits result from short-lived but steady volcanic plumes, and thus rather intense eruptions.

As it is currently impossible to clearly distinguish in the field the precise origin of each of the measured deposit layers (i.e., the deposits of the concomitant volcanic edifices are interspersed) and as these eruptions are considered close both in space and time^27^, we chose to calculate the average thickness on each site (purple numbers on Fig. 2, in cm). We also show the minimum (blue) and maximum (red) measured thicknesses. The complete outcrop and thickness database is given in Table S1. From field observations, we were able to draw the 50 cm isopach (black line in Fig. 2), which corresponds to the iso-thickness line on which the deposit is 50 cm thick. In the Discussion section, we use this fieldwork data to propose a best-fit eruptive scenario in Petite-Terre and compare it to the simulated hazard maps presented in the following section.

## Results

### Single scenario simulations

**Fig. 3:**
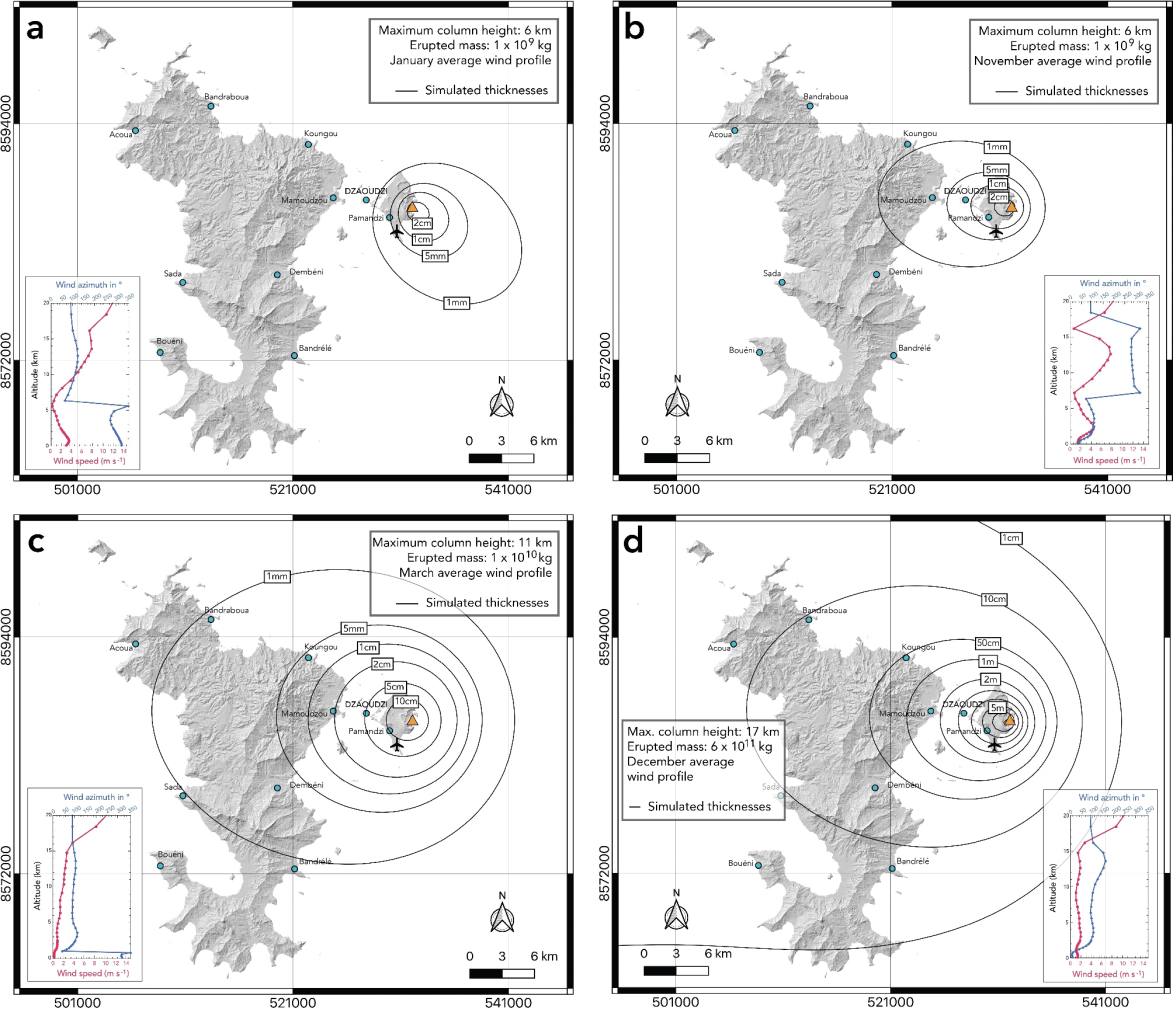
Single scenario simulations from the HAZMAP model for **a)** and **b)** scenario 1 (~ 3 h), **c)** scenario 2 (~ 2h30), and **d)** scenario 3 (~ 28 h, see Methods). The black lines represent isopachs (i.e., iso-thicknesses) simulated by the model. The monthly wind profiles (averaged over 1979 $-$ 2021, see Figure S3 for the complete wind database) used for the simulations are given in insets: January, November, March, and December for **a)**, **b)**, **c)**, and **d)**, respectively. In all simulations, we used a synthetic total grain-size distribution, with a unimodal distribution centered on 2 $\phi$, and based on field data for hydrovolcanic eruptions in the IVESPA database (Figure S2c).

The 36 single scenario simulations (3 eruptive scenarios $\times$ 12 monthly averaged wind profiles over the 43-year database) are shown in movies S1, S2 and S3 of the supporting information. From these simulations, we identified four scenarios (i.e., a pair of one eruptive scenario simulated with one monthly average wind profile) of various impacts for the population of Mayotte, from the less impacting one to the most impacting for each of the three eruptive scenarios. The simulated isopach (i.e., iso-thickness) maps are shown in Fig. 3. As this is the very first volcanic hazard study in Mayotte, we chose this approach to allow a rapid and global vision of the links between the eruptive scenario and the dominant winds at the time of the eruption and their consequences in this region. The maps presented in Fig. 3 therefore do not have the purpose of becoming final hazard maps on which risk studies and evacuation plans could be designed, but rather to be a preliminary step before moving towards a more sophisticated hazard map integrating different volcanic hazards.

We identified a first scenario impacting only Petite-Terre island (Fig. 3a), when considering the eruptive scenario 1 (i.e., a maximum column height *H* = 6 km, and a total erupted mass TEM = 1 $\times$ 10^9^ kg) together with the January average wind profile (inset in Fig. 3a). In this scenario, we simulated a southeastward dispersal axis (consistent with the northwesterlies characterizing the low troposphere in January) with a maximum thickness of 2 cm close to the eruptive vent, while the 1 mm isopach encompasses almost the entire Petite-Terre island. Such a scenario, which we identified as the one with the lowest impact, could already directly threaten more than 29,000 people living on Petite-Terre^28^. The 1 mm isopach covering most of the island also indicates that the airport would likely have to close in case of such an event (see Methods, Table 1). Even in this low impact scenario, because of the location of the drinking water production plant within the 1 cm isopach, water contamination would happen immediately.

**Table 1:** Dry tephra thickness thresholds considered in this study, with their corresponding dry and wet load thresholds, and damages on infrastructures, adapted from^36–43^.

Equivalent dry thickness (m)	Dry mass load (kg m^−2^)	Dry static load (kPa)	Damages to infrastructures	Wet mass load (kg m^−2^)	Wet static load (kPa)
> 0.001	1.02	0.01	Maintenance required on supply networks; reduced visibility; airport closure	1.53	0.02
> 0.01	10.2	0.1	Damaged vegetation, extensive repair on supply networks; contaminated water; breathing difficulties; reduced car speed	15.3	0.2
> 0.05	51	0.5	Long term disruption of wastewater network; extensive damage to most critical components	77	0.8
> 0.10	102	1.0	Replacement required on supply networks; roads impassable for some vehicles	153	1.5
> 0.13	133	1.3	Significant burial of the airport	199	2.0
> 0.20	204	2.0	Roads impassable for all vehicles; collapse of roofs made of timber	306	3.0

Supply networks stand for water and electrical supply and include wastewater network.

Figure 3b shows the isopach map simulated by HAZMAP when considering the same eruptive scenario 1 as before but associated to the November average wind profile (inset in Fig. 3b). In that case, the easterlies characterizing this transitional month drastically change the dispersal axis, threatening not only Petite-Terre island but also the densely populated area of Mamoudzou located on Grande-Terre island where more than 70,000 inhabitants live^28^. Petite-Terre would be impacted by 1 cm of tephra, a thickness threshold corresponding to the airport closure, but also to water contamination (Table 1). The area of Mamoudzou could receive up to 1 mm of tephra, meaning that maintenance would be required on supply networks and that visibility would be reduced on evacuation roads. As most of the critical infrastructures are in Mamoudzou (prefecture, main hospitals, fire stations and schools), even a low tephra thickness could disrupt public services and complicate crisis management.

We then identified a higher impact scenario when considering eruptive scenario 2 (i.e., *H* = 11 km, and TEM = 1 $\times$ 10^10^ kg) together with the March average wind profile (inset in Fig. 3c). The isopach map resulting from the simulations is presented in Fig. 3c. First, we note that the westward dispersal axis of the isopachs is consistent with the easterlies largely dominating the troposphere above 1 km (inset in Fig. 3c). We simulated thicknesses > 10 cm at the eruptive vent, and most of Petite-Terre island would then be impracticable as this thickness threshold corresponds to roads impassable for some vehicles (Table 1). The Mamoudzou area would be impacted by thicknesses ranging from 1–2 cm, and about 2/3 of Grande-Terre island could receive at least 1 mm of tephra.

The last scenario is the most impacting. It simulates the eruptive scenario 3 (i.e., *H* = 17 km, and TEM = 6 $\times$ 10^11^ kg) together with the December average wind profile (inset in Fig. 3d). The isopach map (Fig. 3d) indicates 6 m of tephra at the eruptive vent and more than 3 m on Petite-Terre, meaning a complete destruction of the area. Mamoudzou would be impacted by 1 to 2 m of tephra, and the whole island of Grande-Terre would be covered by more than 1 cm. This scenario is considered very unlikely as it occurred only in two occurrences in the IVESPA database (spanning the 1902 $-$2016 period): the powerful phreatomagmatic phases during the eruptions of El Chichon in 1982 and of Grimsvötn volcano on May 21, 2011^44^.

These four scenarios thus already picture the strong volcanic hazard related to tephra fallout threatening the islands of Mayotte and highlight the vulnerability of the most populated areas (Petite-Terre and Mamoudzou). The population of Mayotte is especially at risk regarding drinkable water, a rare resource on the island that could be contaminated even when considering one of the lowest impacting scenarios.

### Probabilistic map

**Fig. 4:**
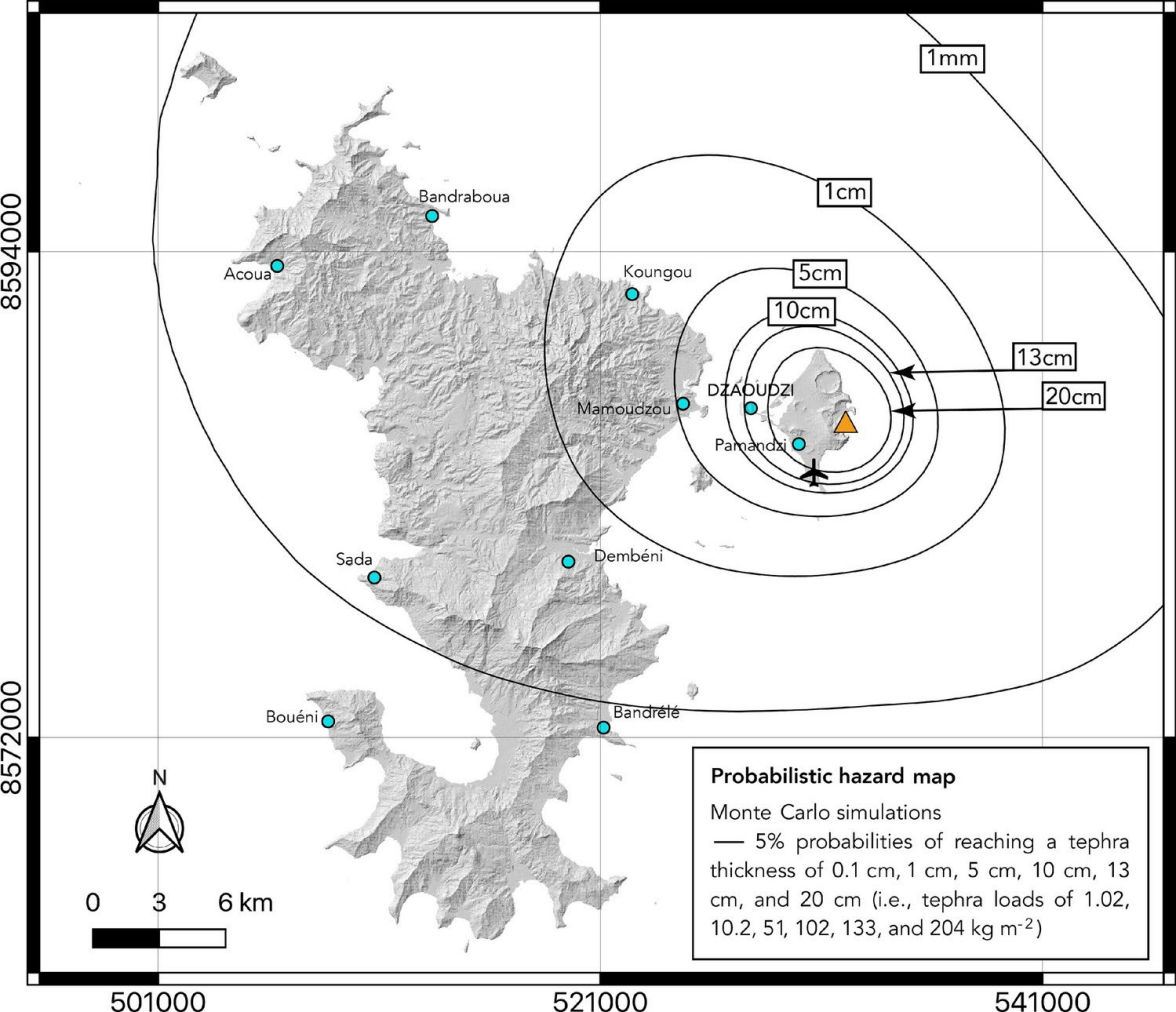
Five% probability map of reaching the different tephra thickness thresholds detailed in Table 1, using a probabilistic approach. We used a Monte Carlo method to randomly select a series of eruptive scenario that we simulated with HAZMAP, the complete 43 years wind database and three different TGSDs (see Methods).

The probabilistic hazard map produced with the Monte Carlo approach is shown in Fig. 4.

As in most of the single scenario maps presented in Fig. 3, the most threatened area by tephra fallout in Fig. 4 remains Petite-Terre island and the highly populated area of Mamoudzou. The easterly trade winds characterizing most of the year greatly impact tephra dispersion, resulting in most material going west from the vent (orange triangle in Fig. 4). However, the high number of simulations performed with the entire wind database (also containing the northwesterly trade winds of the wet season) modulate the main dispersal axis now oriented to the northwest (Fig. 4), compared to what we observed in Fig. 3. The contour of the 20 cm threshold encompasses almost entirely Petite-Terre, while Dzaoudzi and Mamoudzou are likely to be impacted by a 13 cm or 5 cm thick tephra fall, respectively. These thickness thresholds correspond to major disruption in Petite-Terre (significant burial of the international airport for example), and to long term disruption in Mamoudzou (Table 1). Finally, the contour of the 1 cm threshold extends from ~ 6 km southwest of Mamoudzou to ~ 3 km west of Koungou, and the 1 mm contour encompasses ~ 2/3 of the island (up to Bandrélé, South of Grande-Terre). Traces could thus be expected on the entire island, which already correspond to crop damage (like observed in Guadeloupe during the 2010 Montserrat eruptions where pineapple plantations were destroyed by ash thickness < 1 mm). This map shows the broadest possible vision of the hazards linked to tephra fall in Mayotte. In the next section, we will discuss the simulations performed in this study regarding field constraints.

## Discussion and conclusions

Hazard maps are commonly based on the eruptive history of the volcano of interest (with a zonation methodology solely geology-historic based, or with a scenario-based modeling considering past eruptions) or, if the eruptive history is incomplete, on the worst-case or most-likely scenario^33^. New extensive field studies conducted by some of the authors of this work have allowed to unravel the eruptive history of Petite-Terre^27^. However, it usually takes a long time to accurately reconstruct the past eruption source parameters of a volcanic region, especially on small islands. We thus chose in this work to assess volcanic hazards linked to tephra fallout by using data from hydrovolcanic eruptions all around the globe and compiled in the IVESPA database^44^. Such a study was crucial as the Volcanic Hazard Index (VHI^45^) of Mayotte (not considering the Fani Maoré eruption that occurred offshore) ranges between 11 and 18 (depending on the chosen hypotheses, see calculation details in Supporting Information). This places Mayotte in Hazard Level II to III (out of III), and in the maximum threat volcano level (red, level III out of III) when also considering the Population Exposure Index (^46^; see the Supporting Information for more details). We however already present here a simulated best-fit eruptive scenario based on the field data retrieved by^27^ and compare it to the hazard maps presented in the Results section.

**Fig. 5:**
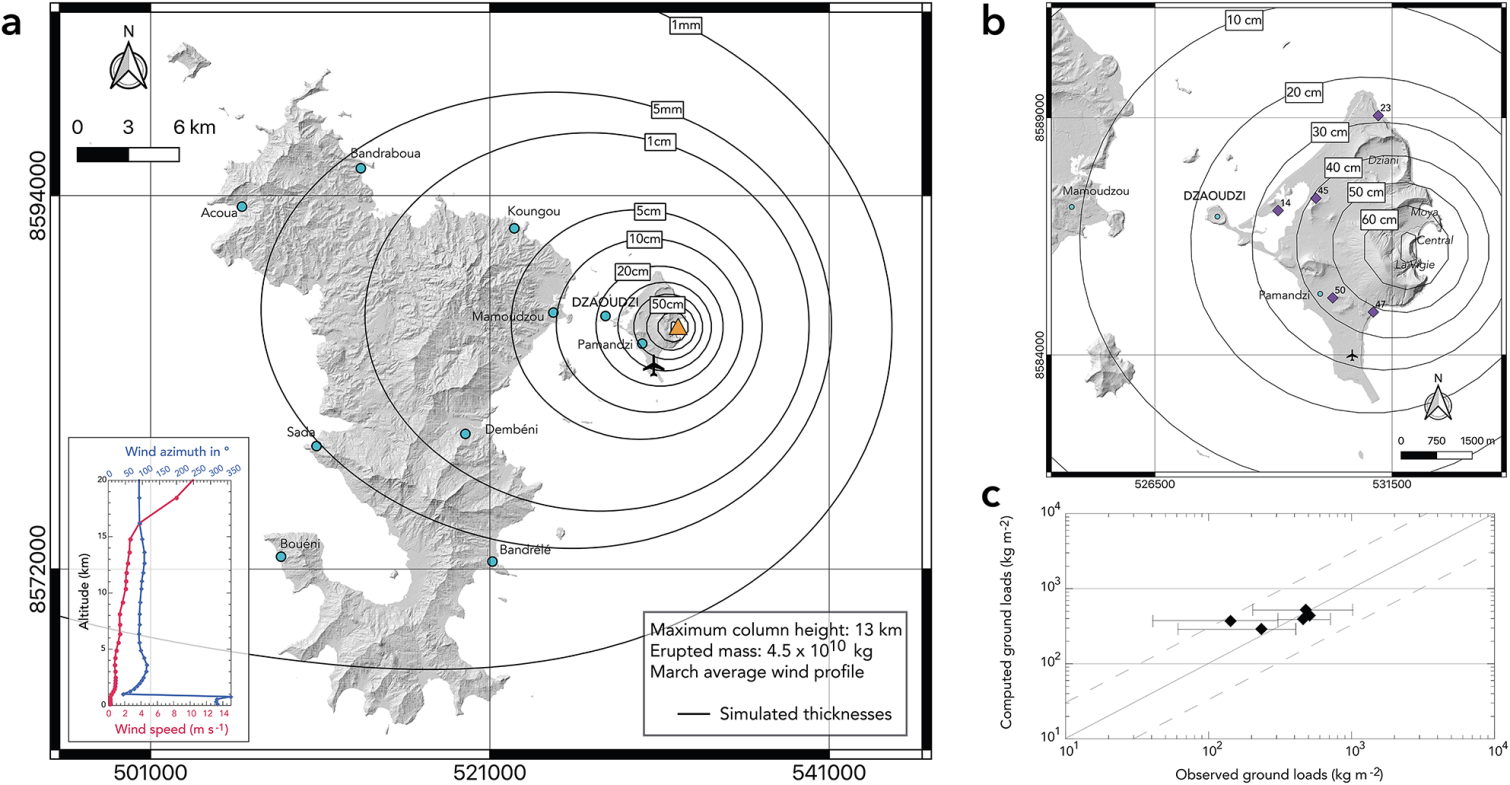
Best-fit simulation for thickness measurements shown in Fig. 2, with (**a)** simulated isopachs (black lines) over Mayotte, (**b) **zoom on Petite-Terre with average thickness measurements from^27^ shown by the purple diamonds and associated values (in centimeters), and (**c)** log–log plot of the observed versus calculated tephra loads (kg m^−2^). Dashed lines in (**c)** indicate over- or under-estimations of 1/3 and 3 times the observed values, respectively. As input parameters for this simulation, we used a maximum column height of 13 km, an erupted mass of 4.5 $\times$ 10^10^ kg, and a March average wind profile for the 1979–2021 period (shown in inset in **a**).

The average thickness of the Fa fallout deposits measured by^27^ allowed us to draw a 50 cm isopach (Fig. 2, see Previous fieldwork in Petite-Terre). Using the method of^47^ that allows estimating a deposit volume from a single isopach, we infer a minimum volume of $\approx$ 0.01 km^3^. Considering the measured deposit density of 1,020 kg m^−3^ (see Methods), this volume yields a minimum mass of tephra emitted of $\approx$ 1.2 $\times$ 10^10^kg, suggesting that these eruptions were at least VEI 3 events^48^. This minimum estimate is fully consistent with our eruptive scenario 2 (Fig. 3c), and comparable to the volumes calculated by^27^ for each edifice (between 0.01 and 0.19 km^3^, and VEI between 2 and 4). Note that in^27^, the erupted volumes were estimated from the crater volumes of the tuff rings and cones, and thus include both fallout and PDCs components, leading to larger volumes than the one we estimated from a single isopach of fallout deposits. In the IVESPA database, an erupted mass from a hydrovolcanic eruption of about 10^10^ kg is associated with a maximum column height between 5.6 and 16 km^44^. We thus expect the best-fit eruptive scenario to be characterized by a column height of around 10 km. We performed a series of single scenario simulations to test several column heights (between 5 and 16 km) and deposit masses (ranging from 10^10^ to 5 $\times$ 10^10^ kg) together with the twelve monthly-averaged wind profiles. In the absence of a reconstructed TGSD from the field (only proximal and limited data on granulometry are available^27^), we used the average TGSD from the IVESPA database (in purple in Figure S2c). The best-fit simulation, obtained for a maximum column height of 13 km, a deposit mass of 4.5 $\times$ 10^10^ kg and the March averaged wind profile is presented as an isopach map in Fig. 5a and b. Figure 5c shows a good consistency between the computed and observed ground loads, which reinforces our confidence in the input parameters used for the simulation.

It is important to remind that this best-fit simulation’s purpose was not to reconstruct the eruptive parameters from field data, as the measured thicknesses are averaged over several explosive events most probably originating from different vents. Instead, we aimed to better capture what would be the most probable (and median) eruptive scenario in the future. Based on average thickness measurements, we note that this scenario is consistent with the impacting scenarios presented in Fig. 3, as it is strongly similar to Scenario 2 (Fig. 3c). This suggests that the explosive eruptions characterizing the Petite-Terre activity were rather powerful, as their ESPs are most likely comprised in the upper half of the ESPs of hydrovolcanic eruptions recorded in the IVESPA database (Figure S2a, b).

**Fig. 6:**
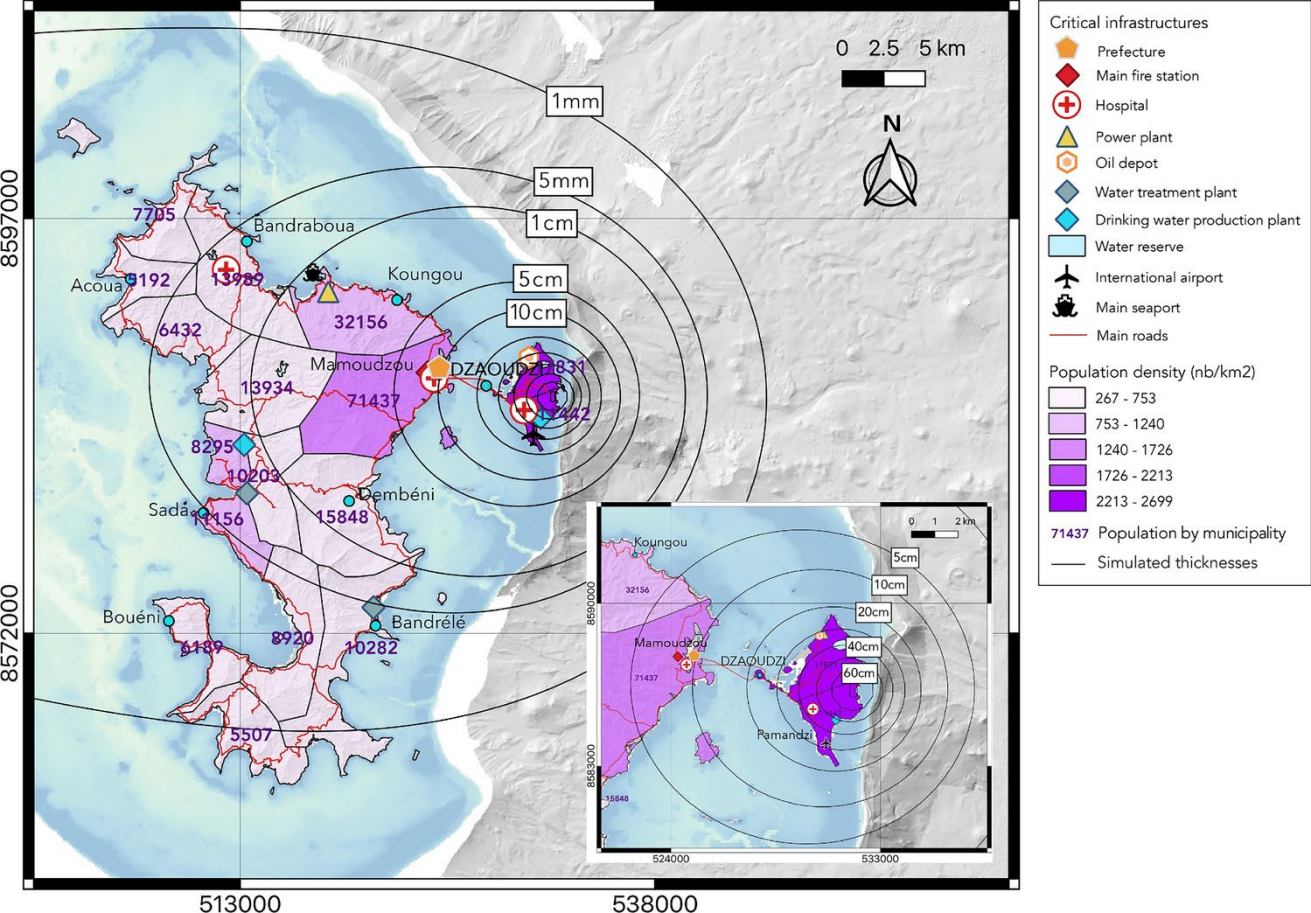
From hazard assessment to a first vulnerability assessment: superposition of the simulated isopachs for the best-fit scenario (Fig. 5) with selected critical infrastructures in Mayotte. The insert shows a closer view of the simulated isopachs on Petite-Terre. Shades of purple show the population density; purple numbers indicate number of inhabitants (from^28^).

Bearing in mind that a full risk assessment is beyond the scope of this paper, we show in Fig. 6 a first confrontation between the best-fit scenario presented in Fig. 5 and selected data on population and critical infrastructures (i.e., physical structures, systemic networks…, that provide essential services to the social and economic functioning of society).

The high vulnerability of Petite-Terre and Mamoudzou to tephra fall is visible as the Prefecture, the international airport, two main hospitals, the main fire station, a water reserve, one of the two thermal power plants, an oil depot, and a drinking water pumping station would be impacted by 5 to 40 cm of tephra. Petite-Terre, where the population density is the highest in Mayotte, could expect more than 20 cm of ash, and would most probably also be subjected to ballistic fall (volcanic rocks with diameters > 4 cm) as demonstrated by field studies^27^. This is of crucial importance for crisis management as the 20 cm thickness threshold corresponds to collapse of roof made of timber (Table 1). As expected, this area should thus be evacuated with the highest priority. The main seaport of the island and the second thermal power plant could be impacted by a 1 cm thick deposit of ash in case of a future explosive volcanic event, leading to extensive repair and breathing difficulties. As this thickness threshold of 1 cm also corresponds to reduced car speed, it is worth noting that the traffic could be considerably complicated in this area. At the same time, we can expect the evacuation routes to be the coastal national roads N1 and N2 that depart from Mamoudzou (Fig. 6). In this case, imposed/directed itineraries by the local authorities towards different pre-defined escape points are crucial as this was proved to be the most effective and safest way to evacuate^49^. Finally, the third main hospital, the second drinking water pumping site and two water treatment plants are located within the 5 mm isopach, meaning long-term disruption of the water networks impacting the whole island, already subjected to high water shortage risks. Based on this map, almost the entire island could receive at least 1 mm of tephra, and an area of 454 km^2^ could be impacted by at least 1 cm of tephra, threatening about 173,000 inhabitants according to the last INSEE census (Institut National de la Statistiques et des Etudes Economiques^28^).

Finally, to guide future thinking about the vulnerability of crucial infrastructures to a volcanic explosive event in Petite-Terre and based on the Monte Carlo simulations presented in Fig. 4, we calculated the probabilities of the chosen crucial infrastructures of being impacted by the first two tephra thresholds (1 mm and 1 cm). The results are presented in Table 2.

**Table 2:** Probabilities of reaching critical thresholds at key locations in Mayotte in case of an explosive volcanic event located on Petite-Terre (Central Crater), according to Fig. 4.

Critical infrastructure	Thickness threshold	
	1 mm	1 cm
Drinking water pumping site of Pamandzi	80.6%	33.1%
Hospital of Petite-Terre	79.2%	32.0%
Thermal power plant of Badamiers	78.4%	29.2%
Oil depot of Badamiers	76.4%	27.1%
International airport of Dzaoudzi-Pamandzi	61.2%	19.1%
Hospital of Mamoudzou	39.3%	11.0%
Prefecture of Mayotte	39.3%	11.0%
Main fire station of Mamoudzou	36.1%	10.3%
Thermal power plant of Longoni	13.6%	4.8%
Main seaport (Longoni)	13.2%	4.6%
Hospital of Dzoumogné	8.6%	3.0%
Drinking water pumping site of l’Ouroveni	7.6%	2.7%
Central water treatment plant	6.6%	2.1%
Water treatment plant of Bandrélé	4.9%	1.3%

We can note that all the chosen crucial infrastructures have a probability $\ge$ 5% of being impacted by a future eruptive scenario (i.e., to receive at least 1 mm of tephra). The probability to reach this first threshold even reach $\approx$80% for one of the drinking water pumping sites and the hospital in Petite-Terre. All infrastructures also have a probability between 1 and 33% to receive at least 1 cm of tephra, the probability is even > 5% for 8 of the 14 infrastructures. These results highlight the need of integrating the scenario of a reactivation of the Petite-Terre eruptive vents into a future emergency plan for Mayotte. While this study will be strengthened in the future thanks to new field data and simulations of ballistics (as in^50^and^51^for Vulcano island and La Soufrière de Guadeloupe, respectively) and by a PDC deposit map (as in^52^), it already allows a more thorough vision of volcanic hazard in Mayotte and can be considered as a baseline study from which moving forward. As the Prefecture of Mayotte is seeking to produce the first emergency plan in case of a volcanic eruption in this island, we also expect the maps presented here to be a powerful communication tool for the local authorities and the population, and help raising public awareness.

## Methods

We used the 2-D tephra dispersal model HAZMAP (^31^, version 2.5.2) together with the ERA5 wind reanalysis database from 1979 to 2021^34^to assess the tephra fallout hazards in Mayotte in case of a reactivation of the Petite-Terre eruptive vents. The HAZMAP model is commonly used to simulate tephra dispersal from small to moderate (i.e., between 3 and 14 km high) volcanic columns^53–56^. We first identified several single eruptive scenarios of different impacts on Mayotte island that we simulated along with a single average wind profile. We then used a Monte Carlo approach to perform probabilistic simulations (i.e., considering randomly selected eruptive scenarios together with the 43-year wind database). Computing single scenario and probabilistic simulations provide complementary information on hazard levels linked to tephra fall^57^. The resulting maps proved to be a crucial communication tool between scientists and local authorities for the design of evacuation routes and for crisis management^42,57^. Note that in all simulations, we considered an eruptive vent located inside the Central Crater, east of Petite-Terre (orange triangle on Figs. 3, 4 and 5), for its central position amongst the monogenetic volcanic complex formed by the La Vigie, Moya, Central Crater and Dziani edifices. Given the proximity between the eruptive centers (from 500 m to 1 km), changing the vent location from the Central Crater to another of these edifices would cause very little variation in the hazard areas.

### Eruption source parameters

Two sets of input parameters are required to run the simulations: eruptive source parameters (ESPs) and wind fields. There are very few examples (like Ruapehu 1996^58^; or Etna 2001^59,60^) in the literature of hydrovolcanic eruptions for which ESPs $-$ maximum column height *H*, total erupted mass (TEM), total grain-size distribution (TGSD) $-$were precisely estimated, as deposits from these eruptions are often of small volume, dispersed over a limited area, and eroded rapidly after the event. Winds, weathering, rainfall or subsequent more violent magmatic phases of the eruption may alter the deposits, preventing a thorough field analysis and reconstruction of the eruptive dynamics. Furthermore, deposits from past eruptions in Petite-Terre originate from four eruptive vents, close both in distance and time, making it even more complex to clearly distinguish the different eruptive phases and estimate their corresponding ESPs^27^.

To tackle the difficulty of the lack of field data in Mayotte, we used the recent IVESPA worldwide database^44^ in which thirty-seven explosive events are reported as hydrovolcanic events (either phreatic or phreatomagmatic). Their column heights and total erupted masses range from 2.3 to 17.5 km and from 10^7^ to 9.6 $\times$ 10^11^ kg, respectively (Figure S2a, b; and Supplementary Table 1 in^57^). We thus consider in this study column heights ranging from 2 to 17 km, and total erupted masses from 10^6^ to 10^12^ kg to account for smaller eruptions that may not be well recorded in the field. Varying these parameters allows reducing the uncertainties on the produced hazard maps, as the TEM was proved to have a first order effect on the extension of hazard areas^55^.

Both grain size and deposit thickness data at multiple outcrops are required to estimate the TGSD of an eruption. Tephra sampling of hydrovolcanic eruptions is currently limited in the literature due to the common highly altered nature of hydrovolcanic deposits. We thus used a synthetic TGSD based on field data for phreatomagmatic and phreatic eruptions in the IVESPA database (Figure S2c). This TGSD has a unimodal distribution centered on 2 $\phi$, with a mean diameter of 1.16 $\phi$ and a sorting $\sigma$ of 2.59. This TGSD corresponds to a median of the twelve best-constrained events in the database. Ideally, the TGSD should incorporate a clast-type distribution (considering both juvenile vesiculated pumices and accidental dense lithics) and thus the different densities of each clast-type, but this detailed information is rarely available in the literature (e.g^40,61^). Quantifying the effect of the TGSD on the hazard maps is not straightforward, and previous sensitivity tests demonstrated that a greater fraction of fine particle led to smaller hazard areas^55^. To consider the possibility for a hydrovolcanic eruption to produce a coarser or finer TGSD depending on the nature of the material removed from the volcanic edifice, we thus also used in the probabilistic simulations two additional TGSDs from the IVESPA database with unimodal distributions centered on 0 $\phi$ and 5 $\phi ,$ with mean diameters of −0,83 $\phi$ and 3,98 $\phi$, and sorting of 2,43 and 2,83, respectively (Figure S2c).

For all simulations, we used a horizontal diffusion atmospheric coefficient of 3,000 m^2^ s^−1^, a value commonly used in the literature^55^. Note that increasing this value up to 5,000 m^2^ s^−1^only leads to a 6% variation of the hazard area^55^. We also parameterized in all simulations the mass distribution of particle in the volcanic column by using two Suzuki parameters set at A = 4 and $\lambda$ = 1, as they represent a ratio ${H}_{B}/{H}_{T}$ (where ${H}_{B}$ is the neutral buoyancy height of the plume, and ${H}_{T}$ its maximum height) similar to the one observed for buoyant plumes^62,63^. Finally, as HAZMAP returns as outputs a grid in which each cell is given a calculated tephra load (in kg m^−2^), we used a dry deposit density of 1,020 kg m^−3^ to convert tephra loads into thicknesses (in cm). This deposit density was measured on three bulk samples from the Fa fallout deposits at the Badamiers beach (BAD2 in Table S1, outcrop site number 2 in Fig. 2). These deposits are described in the section dedicated to previous fieldwork in Petite-Terre.

### Meteorological dataset

The HAZMAP model requires wind profiles, including wind velocity components (*u*, *v*) as a function of altitude. We used wind fields from the European Centre for Medium-Range Weather Forecasts ERA5 reanalysis for the 1979 $-$2021 period^34^. The initial content of ERA5 files consists of hourly global fields of zonal and meridional wind data (direction, velocity) at a horizontal resolution of 0.25° $\times$ 0.25° ($\approx$ 31 km) and vertically distributed on 37 pressure levels from 110 m (1000 hPa) to $\approx$48 km (1 hPa). These wind fields have been interpolated to match HAZMAP format by converting each of the 37 pressure levels into an altitude level using an altitude model (Figure S1 in^42^). We selected the wind components over Mayotte at each time step and each pressure level in an area ranging from 12.8°S to 12.9°S and from 45.3°E to 45.4°E, and we calculated a mean daily wind profile for every day from January 1, 1979 to December 31, 2021. Our final dataset is thus composed of 15,706 vertical wind profiles (365 or 366 days times 43 years), as shown in Figure S3. From these daily wind profiles, we also calculated twelve monthly averaged wind profiles (one for each month), used for the single scenario simulations.

Mayotte is characterized by two main seasons: a wet season (Kashkasini) from December to March, and a dry season (Kussini) from June to September. They alternate with two interseasons, Matulahi in April–May and M’Gnombéni in October–November (Figure S3). These seasons are linked to two main wind regimes in the lower troposphere (< 7 km) in Mayotte: the hot and wet north-northwesterly winds during the austral summer (Figure S3a), and the dry and colder southeasterly trade winds produced by the Mascarene anticyclone during the austral winter (Figure S3e). The interseasons correspond to transitional months between the two mains regimes with southeasterlies during Matulahi (Figure S3c), and east-northeasterlies during M’gnombéni (Figure S3g). While tephra dispersal is mainly controlled by low-tropospheric trade winds, wind regimes characterizing the mid and upper-troposphere (up to 17 km in tropical regions) can occasionally influence the dispersal axis, especially if the trade wind speed is low^64^. Winds in the mid and upper troposphere are often called “antitrade winds” as a change in speed and direction occurs around 5 $-$ 7 km. This change is quite visible in Figure S3 where southeasterlies in the upper troposphere characterize the wet season (Figure S3b), while northwesterlies generally occur during the rest of the year (Figure S3 d, f, h).

### Single scenario simulations

We first performed single scenario simulations based on the historical record of hydrovolcanic eruptions in the IVESPA database (Figure S2). For these simulations, we considered three scenarios with a column height of 6 km and a TEM of 1 $\times$ 10^9^ kg (scenario 1), a column height of 11 km and a TEM of 1 $\times$ 10^10^ kg (scenario 2), and a column height of 17 km and a TEM of 6 $\times$ 10^11^ kg (scenario 3). Each of the three eruptive scenarios was then tested against twelve monthly wind profiles averaged over the period 1979 $-$ 2021 to identify scenarios of various impacts for the population in Mayotte, presented in the Results section (Fig. 3).

### Probabilistic approach

We used a Monte Carlo method to generate probabilistic maps including the variability of both eruptive scenarios and possible wind conditions. For the maximum column height and the TEM, we assumed normal distributions whose maximum and minimum values of each parameter are determined from historical hydrovolcanic eruptions compiled in the IVESPA database (^44^, see Figure S2a, b). For the maximum column height, we assumed a normal distribution with a mean value of 7.5 km and a standard deviation of 2.5, and then randomly selected values. For the erupted mass, the lognormal distribution was centered on 9 (i.e., 10^9^ kg) with a standard deviation of 1, and we then randomly selected values. Each pair of maximum column height and total erupted mass was then combined with three different TGSDs (described earlier in the Methods section). In total, 565,416 simulations were made using the complete ERA5 wind database. The results are presented in Fig. 4 using the six reference tephra thickness thresholds (see below).

### Tephra load thresholds

HAZMAP requires tephra load thresholds as additional input information in order to compute maps showing the probabilities to reach each of the chosen thresholds in every part of the considered region. Choosing six tephra load thresholds thus corresponds to generating six probability maps in which each grid cell is given a probability to equal or exceed the considered threshold. We selected six reference dry tephra thickness thresholds corresponding to different tephra thicknesses and degrees of damage on vegetation, infrastructures, or networks (Table 1) derived from the literature and empirical data: 20 cm, 13 cm, 5 cm, 1 cm, and 1 mm^36–43^. The 20 cm threshold corresponds to heavily damaged roads that prevent vehicles to pass, and collapse of roofs made of timber^36,38–41,43,65^. The 13 cm threshold corresponds to a significant burial of the airport (the complete burial being set a 15 cm by^41^) and to the last limit before roof collapse^36,38–40^. A thickness of 10 cm provokes widespread disruption, requires replacement of supply networks, and makes roads impassable for some vehicles^41,43^. The 5 cm threshold corresponds to long term to possible permanent disruption of the wastewater network and extensive damage to most critical components contained in heating, ventilation, air conditioning or computerized systems^41^. When 1 cm of ash thickness is reached, vegetation and crops are likely to be damaged and extensive repair is required on supply networks^37,40,41^. At this stage, water is likely to be contaminated, breathing difficulties can occur, and car speed is markedly reduced^41^. Finally, the 1 mm threshold corresponds to maintenance required on supply networks (including water and electrical supply, and wastewater network), reduced visibility and airport closure^40–42^.

Depending on the deposit density considered (1,020 kg m^−3^in this study), each thickness threshold corresponds to a different dry mass load threshold implemented in HAZMAP. To allow the tephra loads to be compared with static load values determined by engineering studies for the vulnerability of roofs and walls to structural failure^38,39^, we also converted the isomass values into values of isostatic maximum load of dry tephra (in kPa) using the same method as^40^ (their Eq 1). The correspondence between thickness, dry mass load thresholds, and dry static load thresholds is summarized in Table 1. Finally, note that these values increase when converted into wet tephra static load considering local rainfall or humidity saturating tephra. Considering wet tephra with a 50% volume saturation by water, the deposit density increases to at least 1,530 kg m^−3^^40,66^. Corresponding wet tephra static load values are given in Table 1. All simulations were made considering the dry mass loads, but one must bear in mind that for a given thickness, the tephra load will be greater when considering tephra saturated by water. In that case, structural damage corresponding to specific load values will occur at lower thicknesses^40^. The probabilistic map in Fig. 4 shows the 5% probability isocontour of reaching each thickness threshold. The 5% probability is commonly used in the literature (e.g^55^, and references therein) as it corresponds to the lower bound of the 95% confidence interval on a log normal distribution of probabilities (the upper bound being taken at 100%). Figure 4 thus depicts all areas most likely to be impacted by a future hydrovolcanic eruption resulting from the reactivation of eruptive centers in Petite-Terre. Table 3 summarizes our methodology to produce the hazard maps presented in this paper.

**Table 3:** Summary of the methodology used in this study.

**Method**	**H** (km)	**TEM** (kg)	**TGSD** (Fig. S2c)	**Wind profile**	**# runs**	**MER** (kg s^−1^)	**Duration**	**Results**
Single scenario	6	1 × 10^9^	Median	Monthly average (12 months)	12	9 × 10^4^	~ 3 h	Figure 3a and b
	11	1 × 10^10^	Median	Monthly average (12 months)	12	1 × 10^6^	~ 2h40	Figure 3c
	17	6 × 10^11^	Median	Monthly average (12 months)	12	6 × 10^6^	~ 28h30	Figure 3d
Probabilistic	2–17	10^6^–10^12^	Fine, median, coarse	1979–2021	565,416	-	-	Figure 4
Best-fit scenario	13	4. 5 × 10^10^	Median	Average March	1	2 × 10^6^	~ 6h15	Figure 5

H = column height; TEM = total erupted mass, TGSD = total grain size distribution. The MER (mass eruption rate) is estimated from the maximum column height, using^67^ (their Table 4, for tropical atmospheric conditions). The hypothetical duration is calculated from the MER and the TEM. All calculations are made for a mass distribution within the eruptive column given by the Suzuki coefficients A = 4 and $\lambda$ = 1, the horizontal atmospheric diffusion coefficient is set at 3,000 m^2^ s^−1^.

## Supplementary Information

Supplementary Video 1.

Supplementary Video 2.

Supplementary Video 3.

Supplementary Information 1.

## Data Availability

All data generated or analysed during this study are included in this published article (and its Supplementary Information files).
