# Supplementary material for: Tephra fallout hazard assessment for a hydrovolcanic eruptive scenario in Mayotte
Source: Sci Rep. 2024 Dec 30;14:31880. doi: 10.1038/s41598-024-83266-x (PMC11686065; doi:10.1038/s41598-024-83266-x)
Supplement: Supplementary file 4 — Supplementary Information 1. [file 41598_2024_83266_MOESM4_ESM.pdf]

Supporting information of "Tephra fallout hazard assessment for a hydrovolcanic eruptive scenario in Mayotte"

Audrey Michaud-Dubuy, Jean-Christophe Komorowski, Tristan Lacombe and Lucia Gurioli

December 9, 2024

Contents of this file

1. Figure S1
2. Table S1
3. Figure S2
4. Calculation details for the Volcanic Hazard Index (VHI) of Mayotte.
5. Figure S3
6. Captions for the movies S1, S2, and S3

Introduction This supporting file provides supplemental informations on field data acquired in Petite-Terre, Mayotte (photographs in Figure S1 and thickness measurements in Table S1), on the IVESPA database used in this work (Figure S2), on the wind regimes in Mayotte (Figure S3), the calculation details for the VHI of Mayotte, and the captions for the movies S1, S2 and S3.

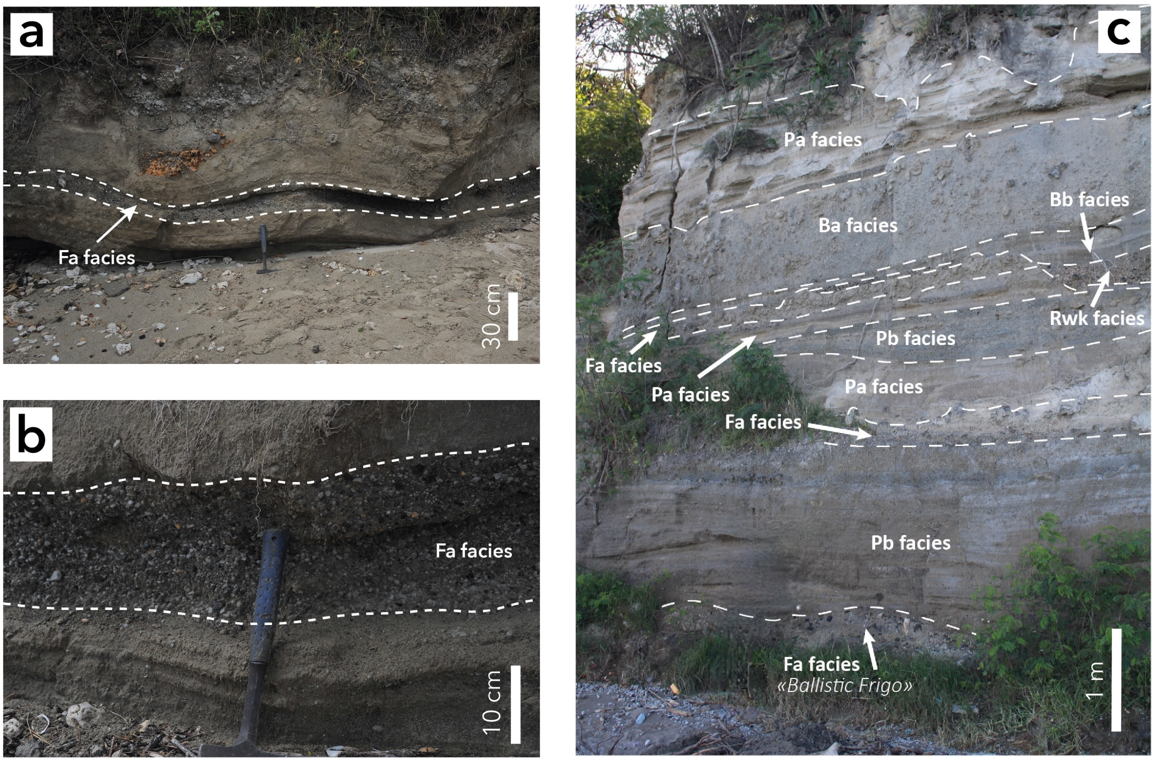

Figure S1: Representative photographs of the different deposit facies observed in Petite-Terre, with **a** and **b** Fa fallout deposits at the Badamiers beach (BAD2 and BAD4 in Table S1), and **c** medial section from La Vigie sequence (ARP3 in Table S1). Scale bars are 10 cm, 30 cm or 1 m long. Boundaries between units are marked by white dashed lines. Modified from Lacombe et al. (2024).

Table S1: Details of the thickness measurements performed by Lacombe et al. (2024) on the five sampling sites presented in Figure 4.

Site	Section	UTM Coordinates		Unit	Thickness (cm)
		East X	North Y		
Vigie	ARP1	531565.431	8585028.858	Ballistic Frigo	20
	ARP2	531102.961	8584905.518	Ballistic Frigo	30
				Fallout 2	30
	ARP3	531075.776	8584857.996	Ballistic Frigo	50
				Fallout 2	30
				Fallout 3	20
	ARP7	530940.988	8584665.725	Ballistic Frigo	100
	ARP8	531016.012	8584791.711	Ballistic Frigo	100
				Fallout 2	40
Dziani	BAD4	531207.184	8589044.076	Fallout 4	10
				Fallout 5	10
	BAD2	530993.683	8589443.525	Fallout 1	6
				Fallout 2	15
				Fallout 3	40
				Fallout 4	30
				Fallout 5	30
				Fallout 6	40
				Fallout 7	30
Mirandola-Mirandolino	MIRA1	529116.065	8587253.172	Fallout 1	16
				Fallout 2	10
				Fallout 3	20
				Fallout 4	30
	MIRA2	529094.789	8587040.759	Fallout 1	4
				Fallout 2	6
				Fallout 3	4
				Fallout 4	6
	MIRO1	529256.22	8587389.047	Fallout 1	20
				Fallout 2	15
				Fallout 3	22
Sandravouangué	SAND1	530247.642	8585203.929	Fallout	50
Totorossa	TOT1	529892.809	8587299.245	Fallout 1	50
				Fallout 2	70
	TOT2	529784.37	8586982.643	Fallout 1	30
				Fallout 2	30

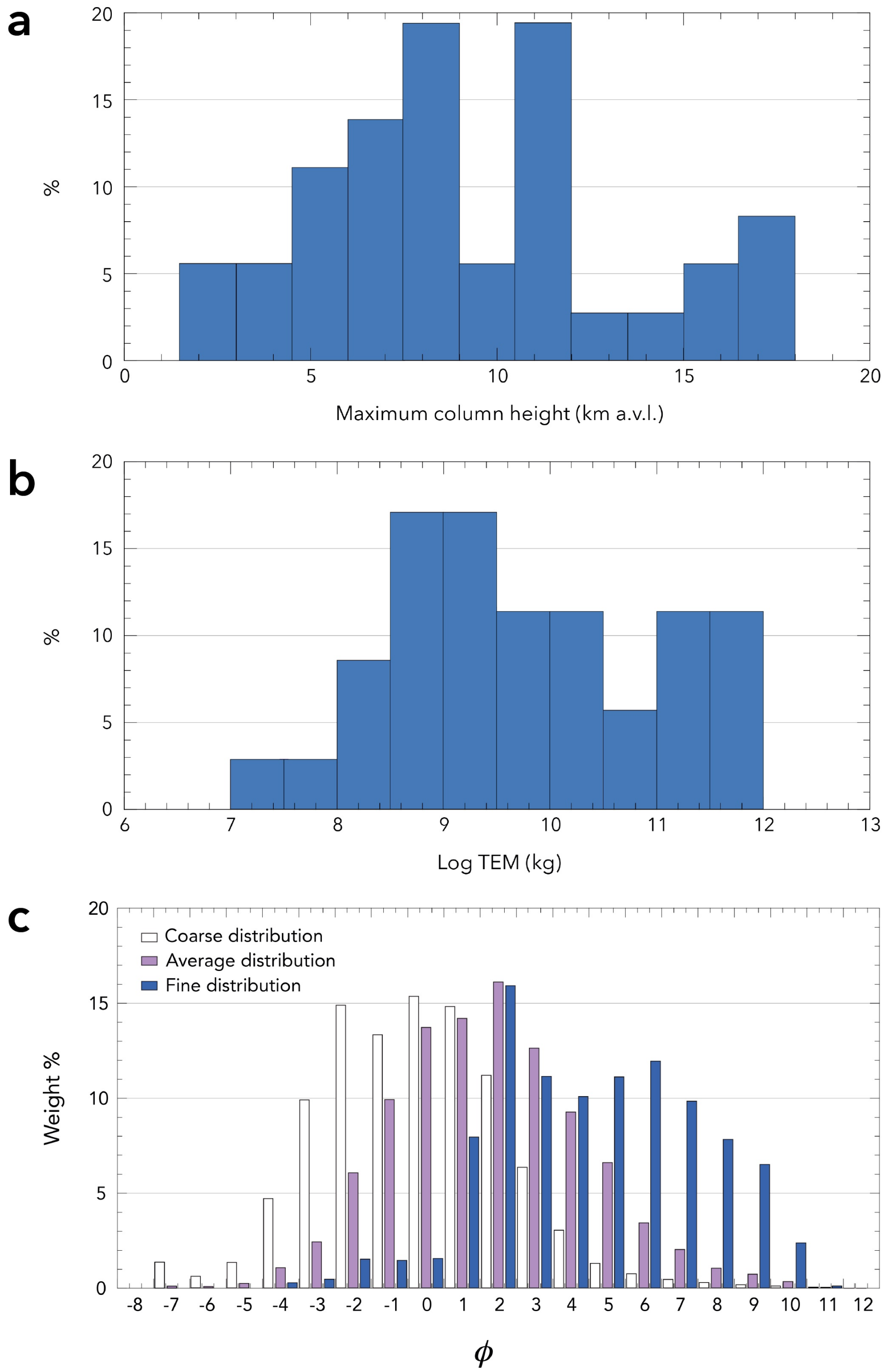

Figure S2: Eruptive source parameters (ESPs) of the 37 phreatomagmatic and phreatic eruptions reported in IVESPA database (Aubry et al., 2021), with **a)** their distribution in maximum column height, **b)** their distribution in total erupted mass, and **c)** the reconstructed total grain-size distributions (TGSDs) used in this study: a median TGSD calculated from the others (in purple), and two additional TGSDs from the database (a coarse distribution in white from the June 17 phase of the Ruapehu 1996 eruption, and a fine distribution in blue from the phase A1 of the El Chichon 1982 eruption).

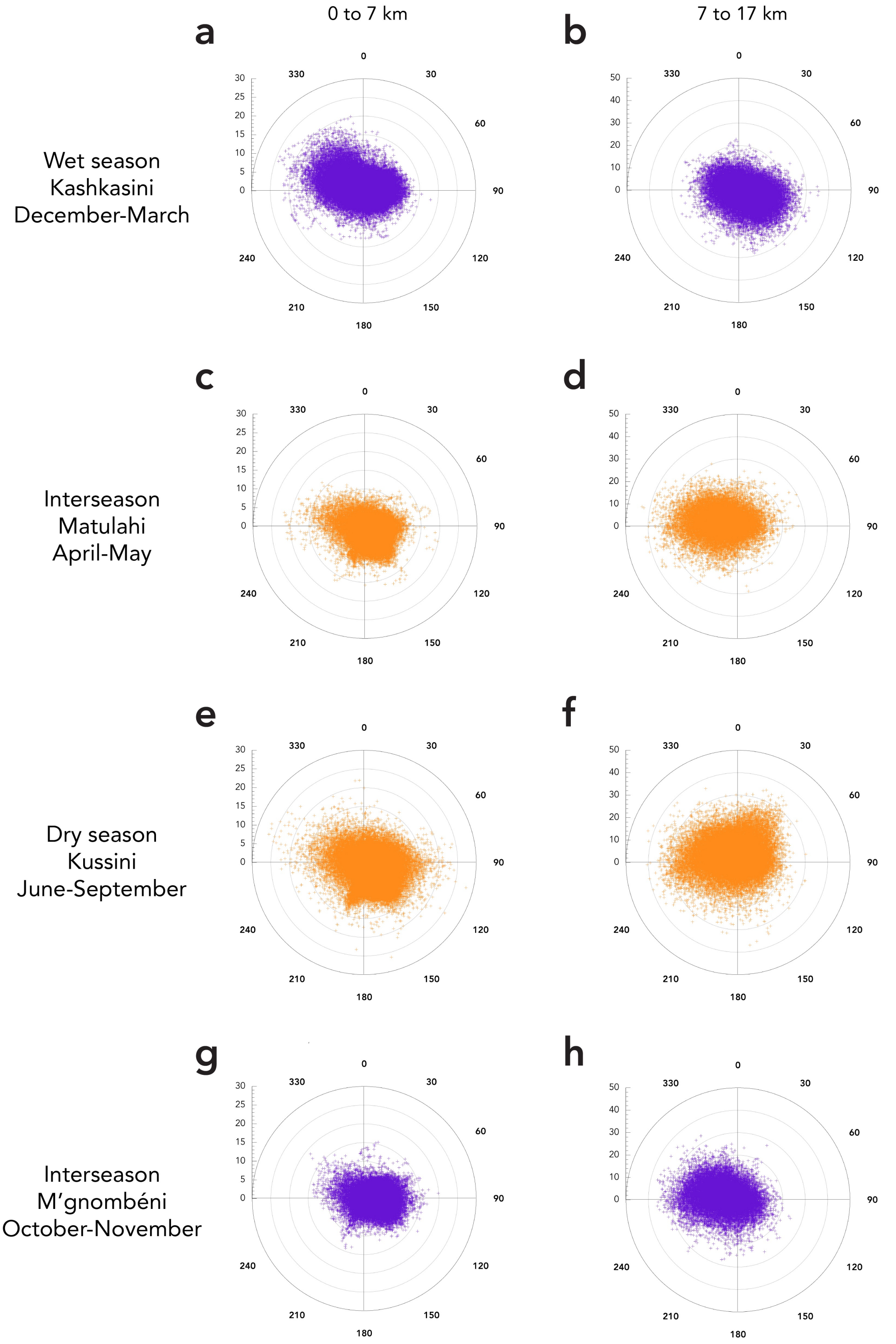

Figure S3: Compass roses representing the 43-year wind database from the European Centre for Medium-Range Weather Forecasts ERA5 reanalysis (Hersbach et al., 2020) for **a)** and **b)** the wet season (Kashkasini), **c)** and **d)** the Matulahi interseason, **e)** and **f)** the dry season (Kussini), **g)** and **h)** the M'gnombéni interseason. Horizontal wind vectors (speed and azimuth) are reported at two intervals of height up to the tropopause: 0 to 7 km (**a**), **c**), **e**), **g**)), and 7 to 17 km (**b**), **d**), **f**), **h**)). The wind speed is discretized into several layers, from 0 m s^{-1} at the center of the rose to the maximum value of each dataset at the rose boundary.

Calculation details for the Volcanic Hazard Index (VHI) of Mayotte

We calculated the VHI for Mayotte, as defined by Auker et al. (2015).

The different parameters used are the frequency status score (1: fully dormant; 1.5: semi-dormant; 2: semi-active; >2: active), the modal VEI within the volcano's counting period, the pyroclastic flow (PF) score (4 if PF are a significant hazard, 0 otherwise), the mudflow (MF) score (2 if MF are a significant hazard, 0 otherwise), the lava flow (LF) score (0.1 if LF are a significant hazard, 0 otherwise), and the maximum recorded VEI.

To integrate the high uncertainty about the eruptive past history of Mayotte, and in a context of very high exposure for the populations, we considered three hypotheses for the calculation of the VHI.

(1) Based only on the eruptions that occurred inland in Mayotte, and considering an age for the last inland eruption of 7.3 ky cal BP (Zinke et al. (2003)), we consider Mayotte to be semi-active (i.e., Holocene eruptions and unrest since 1900CE), giving a frequency status score of 2.

(2) Based only on the eruptions that occurred inland in Mayotte, and considering that the Petite-Terre eruptions are >25 ky old (Lacombe et al. (2024)), we consider Mayotte to be semi-dormant (i.e., no Holocene eruptions, but unrest since 1900CD), giving a frequency status score of 1.5.

(3) We also consider a minimum scenario for which no Holocene eruptions occurred and no unrest directly related to a potential reactivation of the inland volcanism was recorded. In that case, Mayotte is fully dormant, giving a frequency status score of 1.

For the three hypotheses, the modal VEI is taken as 3, the PF score as 4, and both the MF and LF scores are considered to be 0. The maximum recorded VEI is 4 (Lacombe et al. (2024)).

Using the formula of Auker et al. (2015):

$$\text{VHI} = [\text{frequency status score} \times (\text{modal VEI} + \text{PF score} + \text{MF score} + \text{LF score})] + \text{maximum recorded VEI},$$

we calculated a VHI score of **18** for hypothesis 1, **14.5** for hypothesis 2, and **11** for hypothesis 3. Mayotte has thus a hazard level II to III (Auker et al. (2015)). Given that we also calculated a minimum Population Exposure Index (Brown et al. (2015)) of 6, Mayotte is characterized by the maximum threat volcano level (red, level III), as demonstrated by the volcano threat matrix in Auker et al. (2015) (their Figure 22.1).

Captions of the Supplementary movies S1, S2, and S3

Movie S1: Single scenario simulations for the scenario 1, using the twelve monthly wind profiles. Scenario 1 is characterized by a maximum column height of 6 km and a total erupted mass of 1×10^9 kg. From this animation, we can see that the January and November simulations respectively depict a minimum and a maximum impact scenario for the population.

Movie S2: Single scenario simulations for the scenario 2, using the twelve monthly wind profiles. Scenario 2 is characterized by a maximum column height of 11 km and a total erupted mass of 1×10^{10} kg. From this animation, we can see that the March simulation depict a maximum impact scenario for the population.

Movie S3: Single scenario simulations for the scenario 3, using the twelve monthly wind profiles. Scenario 3 is characterized by a maximum column height of 17 km and a total erupted mass of 6×10^{11} kg. From this animation, we can see that the December simulation depict a maximum impact scenario for the population.

From these 36 single scenario simulations, we thus selected the four scenarios of increasing impacts presented in Figure 3.

References

T. J. Aubry, S. Engwell, C. Bonadonna, G. Carazzo, S. Scollo, A. R. Van Eaton, I. A. Taylor, D. Jessop, J. Eychenne, M. Gouhier, L. G. Mastin, K. L. Wallace, S. Biass, M. Bursik, R. G. Grainger, A. M. Jellinek, and A. Schmidt. The Independent Volcanic Eruption Source Parameter Archive (IVESPA, version 1.0): A new observational database to support explosive eruptive column model validation and development. *J. Volcanol. Geotherm. Res.*, 417:107295, 2021. doi: 10.1016/j.jvolgeores.2021.107295.

M.R. Auker, R.S.J. Sparks, S.F. Jenkins, W. Aspinall, S. K. Brown, N.I. Deligne, G. Jolly, S. C. Loughlin, W. Marzocchi, C.G. Newhall, et al. Development of a new global Volcanic Hazard Index (VHI). In S.C. Loughlin, R.S.J. Sparks, S.K. Brown, S.F. Jenkins, and C. Vye-Brown, editors, *Global volcanic hazards and risk*, pages 349–358. Cambridge University Press, 2015.

S. K. Brown, M.R. Auker, and R.S.J. Sparks. Populations around Holocene volcanoes and development of a Population Exposure Index. In S.C. Loughlin, R.S.J. Sparks, S.K. Brown, S.F. Jenkins, and C. Vye-Brown, editors, *Global volcanic hazards and risk*, pages 223–232. Cambridge University Press, 2015.

H. Hersbach, B. Bell, P. Berrisford, S. Hirahara, A. Horányi, J. Muñoz Sabater, J. Nicolas, C. Peubey, R. Radu, D. Schepers, A. Simmons, C. Soci, S. Abdalla, X. Abellan, G. Balsamo, P. Bechtold, G. Biavati, J. Bidlot, M. Bonavita, G. De Chiara, P. Dahlgren, D. Dee, M. Diamantakis, R. Dragani, J. Flemming, R. Forbes, M. Fuentes, A. Geer, L. Haimberger, S. Healy, R. J. Hogan, E. Hólm, M. Janisková, S. Keeley, P. Laloyaux, P. Lopez, C. Lupu, G. Radnoti, P. de Rosnay, I. Rozum, F. Vamborg, S. Villaume, and J.-N. Thépaut. The ERA5 global reanalysis. *Q. J. R. Meteorol. Soc.*, 146:1999–2049, 2020. doi: 10.1002/qj.3803.

T. Lacombe, L. Gurioli, A. Di Muro, E. Médard, C. Berthod, P. Bachèlery, J. Bernard, L. Sadeski, P. Besson, and J.C. Komorowski. Late Quaternary explosive phonolitic volcanism of Petite-Terre (Mayotte, Western Indian Ocean). *Bull. Volc.*, 86(2):11, 2024. doi: https://doi.org/10.1007/s00445-023-01697-2.

J. Zinke, J.J.G. Reijmer, B.A. Thomassin, W-C. Dullo, P.M. Grootes, and H. Erlenkeuser. Postglacial flooding history of Mayotte lagoon (Comoro archipelago, southwest Indian Ocean). *Mar. Geol.*, 194(3-4):181–196, 2003.
